# Engineering stem cell exosomes promotes the survival of multi-territory perforator flap in diabetes via regulating anti-inflammatory and angiogenesis

**DOI:** 10.1093/rb/rbaf075

**Published:** 2025-07-24

**Authors:** Chao Sun, Junwei Su, Zheng Wang, Changjiang Liu, Xinzeyu Yi, Weimin Chen, Dong Zhang, Aixi Yu

**Affiliations:** Department of Orthopedics Trauma and Microsurgery, Zhongnan Hospital of Wuhan University, Wuhan, Hubei 430071, China; Department of Orthopedics Trauma and Microsurgery, Zhongnan Hospital of Wuhan University, Wuhan, Hubei 430071, China; Department of Orthopedics Trauma and Microsurgery, Zhongnan Hospital of Wuhan University, Wuhan, Hubei 430071, China; Department of Orthopedics Trauma and Microsurgery, Zhongnan Hospital of Wuhan University, Wuhan, Hubei 430071, China; Department of Orthopedics Trauma and Microsurgery, Zhongnan Hospital of Wuhan University, Wuhan, Hubei 430071, China; Department of Orthopedics Trauma and Microsurgery, Zhongnan Hospital of Wuhan University, Wuhan, Hubei 430071, China; Department of Orthopedics Trauma and Microsurgery, Zhongnan Hospital of Wuhan University, Wuhan, Hubei 430071, China; Department of Orthopedics Trauma and Microsurgery, Zhongnan Hospital of Wuhan University, Wuhan, Hubei 430071, China

**Keywords:** multi-territory perforator flaps, antioxidation, anti-inflammatory, angiogenesis, engineering exosomes, metformin delivery

## Abstract

The versatile multi-territory perforator flap remains a cornerstone of reconstructive surgery for diabetic ulcerations, yet its clinical efficacy faces significant challenges in hyperglycemic conditions. The diabetic milieu significantly exacerbates tissue ischemia through augmented chronic inflammation and impaired angiogenesis, which collectively harm flap perfusion and compromise its overall viability. A major postoperative complication is distal flap necrosis, which is closely associated with the critical “Choke zone,” a hypoperfused transitional area that exhibits delayed vascular recruitment and suboptimal angiogenesis. This vascular bottleneck creates a precarious balance between tissue oxygen demand and supply, ultimately compromising flap viability. To address this issue, we have developed the engineering stem cell exosomes by encapsulating metformin-loaded Mesoporous silica nanoparticles into BMSC exosomes (M-MS@EXO NPs), enabling the release of metformin. Compared to traditional oral medication, delivering metformin through engineered exosomes allows for precise administration in diabetic wounds. The multifunctional M-MS@EXO NPs exhibit dual pharmacological activity by reducing the secretion of inflammatory cytokines while effectively remodeling the vascular niche within the diabetic microenvironment. Additionally, the M-MS@EXO NPs show anti-inflammatory and angiogenesis effects by inhibiting TNF/apoptosis and enhancing VEGF signaling pathways in vitro. In the dorsal multi-territory perforator flap model of type 2 diabetes, the M-MS@EXO NPs demonstrate the ability to alleviate inflammation and promote neovascularization of the Choke zone, reducing distal necrosis, which holds great promise for improving flap survival in diabetes.

## Graphical abstract

**Figure:**
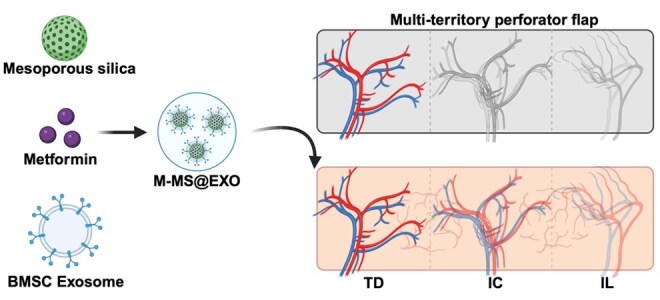

## Introduction

Global statistics indicate that approximately 25% of the 500 million diabetes sufferers will develop localized diabetic foot ulceration during their lifespan, posing significant public health challenges [1–3]. These chronic ulcerations progress into extensive dermal defects that predispose patients to persistent infections and systemic complications. In managing large-scale diabetic wounds, autologous multi-territory perforator flaps have emerged as essential reconstructive tools for achieving volumetric restoration and aesthetic reconstruction within critical timeframes [4, 5]. The growing adoption of this technique in chronic diabetic wound management reflects its clinical advantages over conventional methods.

Perforator flaps are axial pattern flaps vascularized by perforating vessels, initially described in 1989 [6]. The concept of the “perforasome” denotes the largest anatomical territory that can be perfused by the arborizing branches of a single perforator vessel [7]. Multi-territory perforator flaps refer to flaps incorporating two or more perforasomes [8–10]. Each perforasome is linked to the adjacent perforasomes through a series of reduced-caliber vessels in Choke zone. However, extensive damage accompanying hyperglycemia microenvironment leads to immune imbalance and vascularization damage in Choke zone [11, 12], which will eventually lead to flap necrosis. Therefore, the key to improving the survival rate of multi-territory perforator flap in diabetic patients lies in correcting the excessive inflammation of Choke zone and improving the vascularization. In recent years, a bundle of research has focused on exploring the therapeutic potential of exosomes in improving flap survival rates among diabetic patients [13–16].

Exosomes, nanosized extracellular vesicles (30–150 nm) with lipid bilayer structures, are universally secreted by cellular systems [17]. These bioactive particles carry essential molecular components including proteins, mRNAs and miRNAs, demonstrating significant therapeutic potential in regenerative medicine [18–20]. Recognized for their inherent advantages as biological delivery vehicles, exosomes exhibit superior biocompatibility combined with unique capabilities such as biomolecular protection, immunological compatibility and exceptional tissue barrier penetration—characteristics that make them ideal candidates for targeted therapeutic delivery [20, 21]. Recent scientific advancements have consequently focused on engineering these endogenous nanocarriers for precision transport of pharmaceutical agents and genetic materials to pathological sites [22–24]. However, natural exosome faces low yield, impurity co-isolation and batch heterogeneity from labor-intensive ultracentrifugation, limiting scalable therapeutic-grade production and a uniform treatment plan may not always achieve satisfactory therapeutic outcomes. Based on these facts, engineering stem cell exosomes have attracted much attention for its advantages of precise payload delivery, enhanced targeting and reduced immunogenicity [25–27].

**Figure 1.**
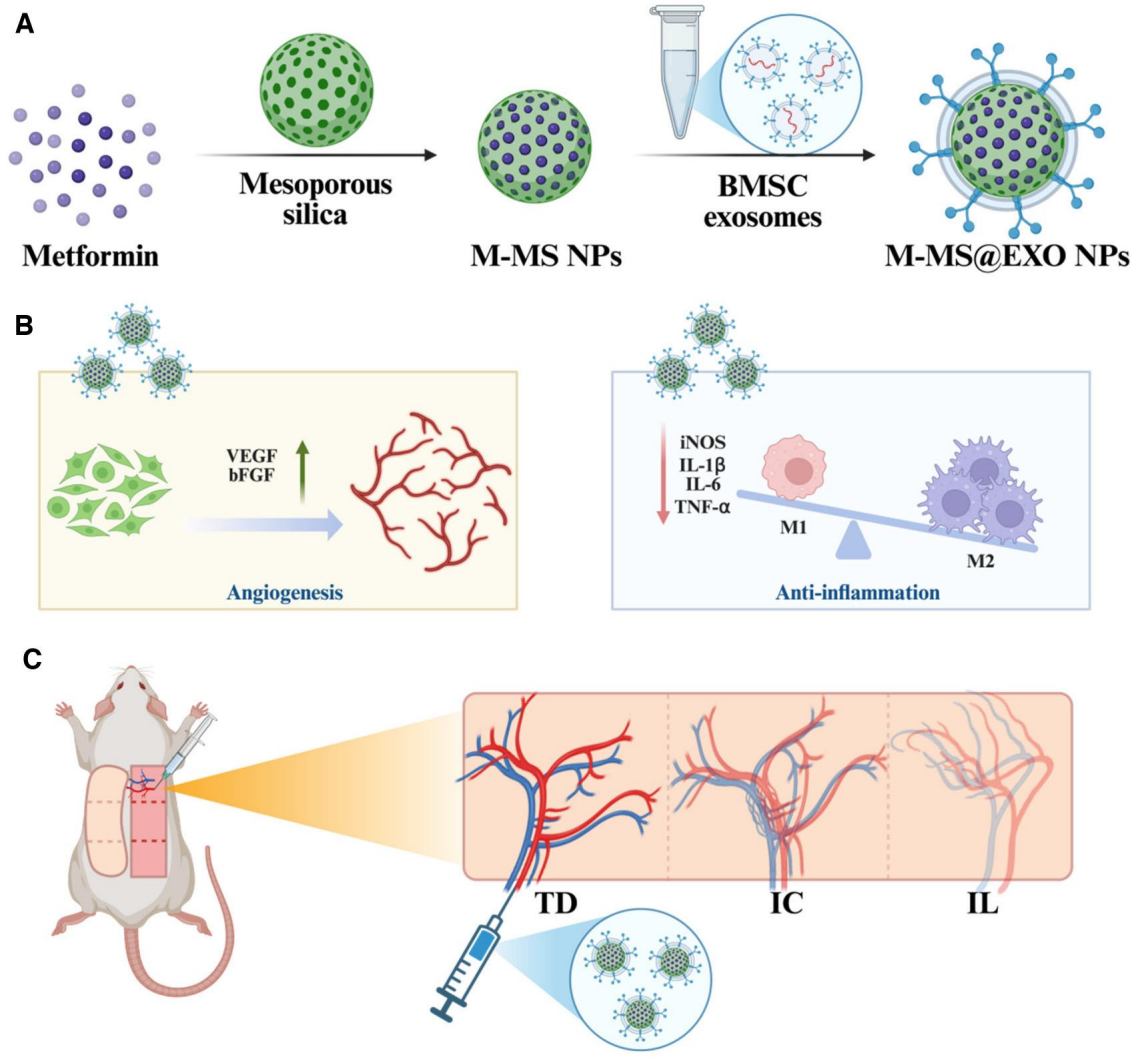
Illustration of engineering stem cell exosomes promotes the survival of multi-territory perforator flap in diabetes via regulating anti-inflammatory and angiogenesis. (**A**) Preparation of the M-MS@EXO NPs. (**B**) The angiogenesis and anti-inflammatory effects of M-MS@EXO NPs. (**C**) The establishment of multi-territory perforator flap in diabetes and *in situ* injection of M-MS@EXO NPs. TD: the thoracodorsal perforator (anatomical zone); IC: the posterior intercostal perforator (dynamic zone); IL: the iliolumbar perforator (potential zone).

Therefore, we designed and developed an engineered exosome (M-MS@EXO) derived from stem cells, incorporating mesoporous silica with metformin to explore the effect on the survival of multi-territory perforator flaps (Figure 1A). Metformin serves as the primary first-line therapeutic agent for type 2 diabetes mellitus management [28]. This pharmacological agent enhances peripheral tissue sensitivity to glucose uptake, thereby reducing systemic hyperglycemia and subsequent oxidative stress in local microenvironments. Furthermore, metformin demonstrates significant microvascular protective properties under hyperglycemic conditions [29], primarily through balancing local excessive inflammation that mediated endothelial damage [30]. Currently, there is a growing interest in using hollow nanoparticles for drug delivery [31]. Mesoporous silica nanoparticles (MSNs) are highly advantageous as drug delivery carriers due to the availability of raw materials, large surface area and porous structure, allowing for efficient loading and controlled release of therapeutic agents. Additionally, their biocompatibility and ease of functionalization enable the targeted delivery of drugs, enhancing therapeutic efficacy while minimizing side effects [32, 33]. In this study, we explore the effects of M-MS@EXO NPs on endothelial cell-mediated vascularization processes and function as well as on the ability to balance excessive inflammation therein *in vitro* (Figure 1B), and whether it could be a potential therapeutic option to relieve the distal necrosis in type 2 diabetes rat model *in vivo* (Figure 1C).

## Materials and methods

RAW264.7 cells and Human umbilical vein endothelial cells (HUVECs) were obtained from the Chinese Academy of Science. Male Sprague Dawley (SD) rat (4 weeks) were purchased from Hubei Bainter Biotechnology Co. The rat bone marrow mesenchymal stem cells (BMSCs) were extracted from the bone marrow cavity of 4-week-old Sprague Dawley rats. Dulbecco’s modified Eagle’s medium (DMEM), specified endothelial cell medium (ECM), penicillin and streptomycin, fetal bovine serum (FBS), PBS, Calcein-AM/PI, staining kits were obtained from Thermo Fisher Scientific. The nitric oxide synthase (iNOS), interleukin-1β (IL-1β), interleukin-6 (IL-6), tumor necrosis factor-α (TNF-α) Elisa kits were sourced from Merck Bioscience Technology Ltd All other standard reagents and experimental consumables were purchased from commercial suppliers (such as Ponsure Biological, Thermo Fisher Scientific) and used without further purification.

### Preparation of M-MS@EXO NPs

CTATOS, TAEHS and ddH_2_O were added to a flask and mixed, then heated to 80°C and stirred for 1 h until fully dissolved. TEOS was quickly added, and the reaction continued at 80°C for 2 h before stopping. After cooling, the mixture was centrifuged and washed several times, then dispersed in ethanol for later use. It was mixed with hydrochloric acid in a specific ratio and stirred at 70°C overnight. The precipitate was collected by centrifugation multiple times to obtain mesoporous silica (MS NPs).

Next, the mesoporous silica was stirred with metformin at room temperature overnight. After centrifugation at 13 000 rpm, the metformin-MS (M-MS) NPs were collected.

Slowly add the BMSCs exosomes extract to the suspension of M-MS NPs, ensuring gentle mixing. Incubate in a 4°C ultrasonic water bath for 5 min, then, warm to 37°C and incubate for a total of 40 min to facilitate the fusion of BMSCs exosomes with the surface of M-MS NPs. Subsequently, purify by ultracentrifugation at 100 000 × *g* at 4°C to separate unbound exosomal components, collecting the precipitate to obtain M-MS@exosome (M-MS@EXO) NPs.

### Characterization

Exosome identification was conducted using TEM (HITACHI, HT7700) to confirm their morphology, DLS (Particle Metrix, Meerbusch, Germany) to analyze diameter distributions and Western blotting to detect specific surface markers, including CD9, CD63 and TSG101.

The change in potential of MS, MS@EXO and M-MS@EXO NPs were determined by zeta potential (Zetasizer Nano ZS90, Netherlands) and the particle sizes were performed by TEM.

### Drug release

The metformin-loaded M-MS@EXO NPs were placed in PBS solution (2 mL). At set time point, solution (100 μL) was taken. The absorbance was measured by a UV spectrophotometer at 235 nm, and the release amount was calculated.

### Live/dead staining

HUVECs were cultivated at a density of 1 × 10^5^ cells per well in 24-well plates in accordance with established protocols. Following the attainment of 90% confluence, the MS, MS@EXO and M-MS@EXO NPs were introduced into the cells for a 24-h period. The viability of the cells was initially determined using a live/dead cell labelling kit (C2015M) containing propidium iodide (PI) and calcein-AM. HUVECs on different hydrogels were stained for 60 min with 500 µl of the composite dye. Fluorescence images were obtained using an inverted fluorescence microscope.

### Cell viability assay

HUVECs were cultivated with the MS, MS@EXO and M-MS@EXO NPs for 24 and 72 h in accordance with previously described methodologies. Subsequently, CCK-8 solution was added to react for 1 h, after which 100 µl of the mixture was pipetted into a 96-well plate. The optical density (OD) at 450 nm was then measured using a microreader (MULTISKAN FC, Thermo Fisher Scientific). The cell viability was then calculated using the following formula: Cell viability percentage = (mean of the treatment group’s absorption value/mean of the control group’s absorption value) × 100%.

### Migration assay

A linear scratch was created on the monolayer cell using a 100-μL sterile pipette tip after the HUVECs had reached 90% confluency. The MS, MS@EXO and M-MS@EXO NPs were then added to the plate. The repopulating/migrated HUVECs were then visualised on the scratches at 0- and 24-h time points. The HUVECs were labelled with calcein-AM and imaged using an inverted fluorescence microscope. The healing ratio of the scratch area was then quantified by means of ImageJ software.

### Matrigel tube formation assay

The Matrigel matrix was utilised to coat 24-well plates (200 µl per well) prior to the seeding of HUVECs (5 × 10^4^ cells/well). The transwell inserts loaded with the MS, MS@EXO and M-MS@EXO NPs were then placed into the plate with caution. The HUVECs were labelled with calcein-AM and observed for tube formation using an inverted fluorescence microscope after a period of 6 h. Subsequent analysis of the parameters of tube formation was conducted using ImageJ software.

### Inflammation and polarization of macrophage in vitro

Lipopolysaccharide (LPS, 500 ng/mL) was used to stimulate RAW 264.7 cells after the cells had reached 90% confluency. Following a 12-h incubation period, the LPS-exposed cells were gently washed with PBS on three separate occasions. The MS, MS@EXO and M-MS@EXO NPs were then gently transferred into the 6-well plates. The LPS group, which was designated as the Control group, was administered an equal volume of PBS. RAW 264.7 cells were then co-cultured with the various samples for a period of 48 h.

#### Immunofluorescence staining

RAW 264.7 cells were subjected to fixation with 4% paraformaldehyde for a duration of 20 min, permeabilization with 0.25% Triton X-100 for a further 20 min and subsequent blocking with 1% bovine serum albumin for a period of 1 h. Thereafter, the cells were subjected to sequential staining with CD206, CD86, Alexa Fluor 488, Alexa Fluor 594 and DAPI. The cells were visualised using a laser scanning confocal microscope. The fluorescence intensities of CD206 and CD86 were then subjected to quantitative analysis using ImageJ software.

#### Quantitative real-time polymerase chain reaction

The total RNA was extracted and reverse-transcribed into complementary DNA using a PrimeScript RT Master Mix. The expression levels of vascular endothelial growth factor (VEGF), basic fibroblast growth factor (bFGF) (from HUVECs), iNOS, IL-1β, IL-6 and TNF-α (from RAW264.7 macrophages) were then examined. Glyceraldehyde-3-phosphate dehydrogenase (GAPDH) was utilized as an internal control gene. Supplementary Table S1 showed the primer sequences of the tested genes.

#### Enzyme-linked immunosorbent assay

The production of cytokines by macrophages was measured by the collection of the supernatants of RAW 264.7 cells. The secretion of the cytokines iNOS, IL-1β, IL-6 and TNF-α was measured by means of commercial ELISA kits, in accordance with the manufacturer’s guidance.

### Transcriptome sequencing

Macrophages suspension (1 × 10^4^ cells/mL) were cultured with various samples in 6-well plates for 72 h. Following this, macrophages were lysed, and the lysates were stored at −80°C prior to RNA sequencing. Gene expression values were transformed to log_10_[TPM (Transcripts Per Million) + 1], and KEGG pathway enrichment analyses were performed using the online platform (www.majorbio.com).

### Establishment type 2 diabetes rat model

The Animal Experimental Ethics Review Committee of Zhongnan Hospital of Wuhan University approved all animal experiments (approval number: ZN2023255). Surgical steps were performed in a sterile environment, and the experimental protocol adhered to animal ethical guidelines. SD rats were were continuously fed a high-fat and high-sugar diet for 4 weeks and intraperitoneally injected with streptozotocin (STZ, 50 mg/kg) dissolved in citrate buffer (pH 4.5). Blood glucose levels were measured every three days. Two weeks after the STZ injection, rats with blood glucose levels above 16.8 mmol/l were considered to be diabetic.

### Establishment multi-territory perforator flap rat model

A multi-territory perforator flap was designed on the right side of the diabetic rat’s back, with the thoracodorsal artery perforating branch positioned as the flap’s tip. The flap encompassed the thoracodorsal artery perforating branch, the posterior intercostal artery perforating branch, the iliolumbar artery perforating branch and the Choke zones I and II. The thoracodorsal artery perforator was preserved, while the posterior intercostal and iliolumbar perforators were ligated. The flap’s dimensions were 11.0 cm × 2.5 cm. Subsequently, 200 μL of solution (containing the MS or M-MS or M-MS@EXO NPs) was injected uniformly into the thoracodorsal artery perforating branch. Following repositioning, *in situ* interrupted sutures were made with medical sutures.

### Flap blood perfusion

The flap images and laser speckle images were taken on Day 0, Day 1, Day 3 and Day 7 after surgery. The laser speckle images were used to measure blood perfusion in the Choke zone II of the flap.

### Pathological staining

The Choke zone II of the flap was subjected to histopathological staining using H&E, Masson’s reagent, as well as immunohistochemistry (VEGF) in the Choke zone II of the flap on Day 7 after surgery and immunofluorescence (TNF-α, IL-10, ROS) in the Choke zone II of the flap on Day 7 after surgery. Fluorescence intensities were quantified using ImageJ software.

### Statistical analysis

The mean ± standard deviation of a minimum of three tests was used to evaluate each experiment. The researchers conducting the interventions and analyzing the results were blinded to the group assignments to reduce bias. Data analysis was performed using GraphPad Prism version 9 software. One-way analysis of variance (ANOVA) was used to analyze data from multiple groups, and two-tailed Student’s *t*-test was used to compare two groups. *P* values of less than 0.05 was considered statistically significant.

## Results

### Preparation and characterization of M-MS@EXO NPs

**Figure 2.**
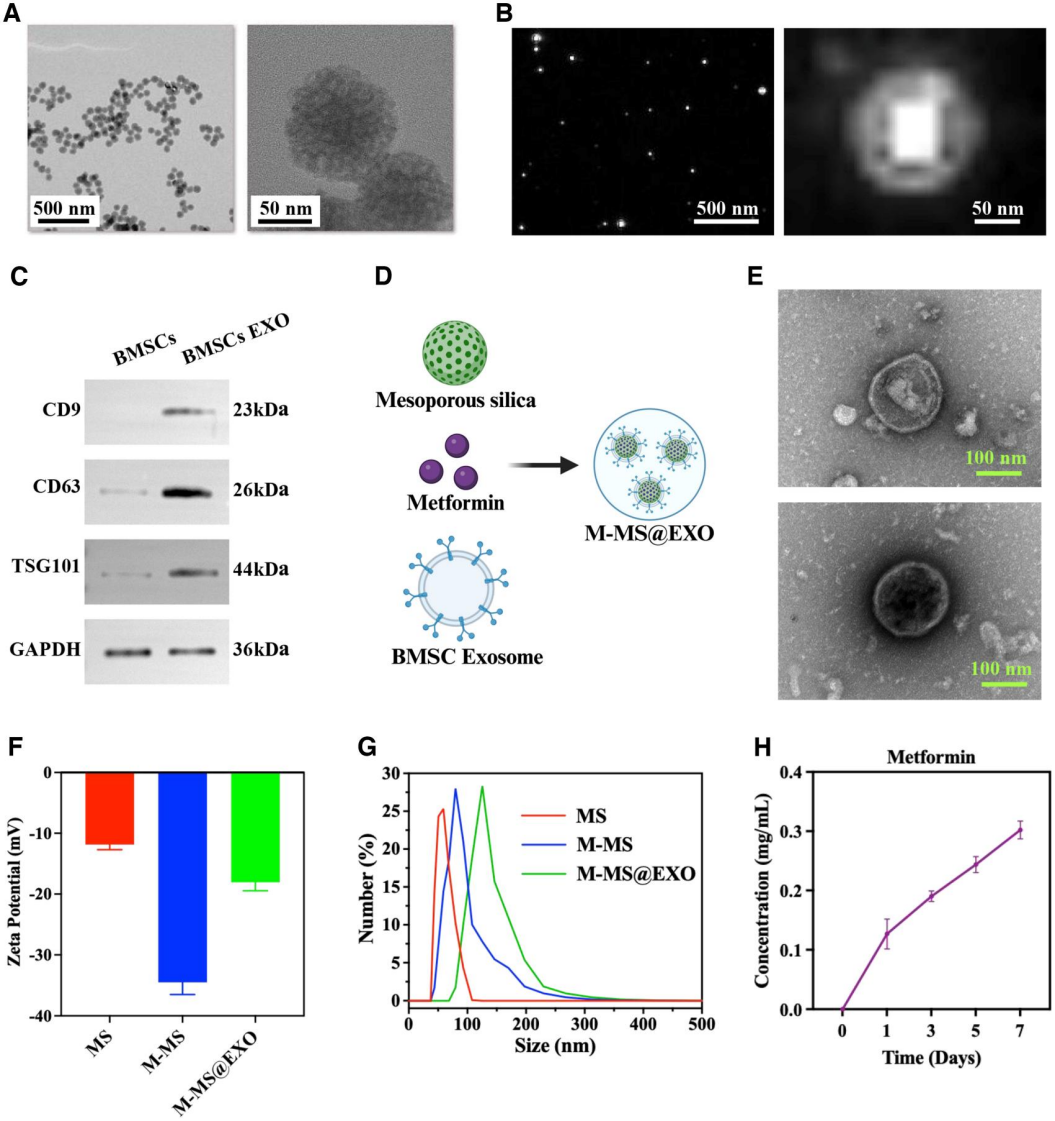
(**A**) TEM images of M-MS NPs. Scale bars: 500 nm, 50 nm. (**B**) DLS images of BMSC exosomes. Scale bars: 500 nm, 50 nm. (**C**) Western blot images of protein expression of CD9, CD63 and TSG101 from BMSCs and BMSCs exosomes. (**D**) Schematic diagram of M-MS@EXO NPs preparation. (**E**) TEM images of BMSCs exosomes and M-MS@EXO NPs. Scale bars: 100 nm. (**F**) Zeta potential patterns of MS, M-MS, M-MS@EXO NPs. (**G**) The particle size of MS, M-MS, M-MS@EXO NPs. (**H**) The cumulative release profile of metformin from M-MS@EXO NPs.

M-MS NPs were obtained by adding metformin into mesoporous silica. TEM images showed the round-morphology of M-MS NPs (Figure 2A). BMSCs were obtained from femurs and tibias of rats, and exosomes were extracted from the BMSCs. Dynamic light scattering (DLS) images showed that the particles displayed a cup- or round- shaped morphology (Figure 2B). The particle sizes measured by DLS analysis varied between 30 and 200 nm (Supplementary Figure S1). Furthermore, the specific surface markers of exosomes including CD9, CD63 and TSG101 were validated by using the Western blotting analyses, supporting the successful isolation of exosomes from BMSCs (Figure 2C).

As shown in Figure 2D, M-MS@EXO NPs, which consisted of mesoporous silica, metformin and BMSCs exosomes, were formed by wrapping BMSCs exosomes around M-MS NPs to create engineered M-MS@EXO NPs. TEM images showing engineered BMSCs exosomes with unencapsulated and encapsulated M-MS NPs, supporting the successful synthesis of M-MS@EXO NPs (Figure 2E). In addition, the zeta potential patterns of MS, M-MS and M-MS@EXO NPs provided crucial insights into their surface charge characteristics, which play a vital role in colloidal stability and interactions with biological systems (Figure 2F). The zeta potential values indicated how the different formulations behave in suspension, with higher absolute values suggesting enhanced stability against aggregation. Moreover, the particle size is a critical parameter that influences the biodistribution and cellular internalization of nanoparticles; smaller NPs may penetrate tissues more easily and are typically better at escaping cellular uptake. The particle size distributions of MS, M-MS and M-MS@EXO NPs showed that exosome encapsulation maintained the nanoparticle size below 200 nm (Figure 2G). The cumulative release profile of metformin from M-MS@EXO NPs showed controlled release behavior over time, which is essential for maintaining therapeutic levels of the drug in systemic circulation (Figure 2H). This sustained release pattern suggested that M-MS@EXO NPs could effectively modulate release kinetics, potentially enhancing pharmacological effectiveness and reducing dosing frequency, particularly advantageous in diabetic conditions where stable drug levels improve outcomes.

### M-MS@EXO NPs promoted the proliferation of vascular endothelial cells

**Figure 3.**
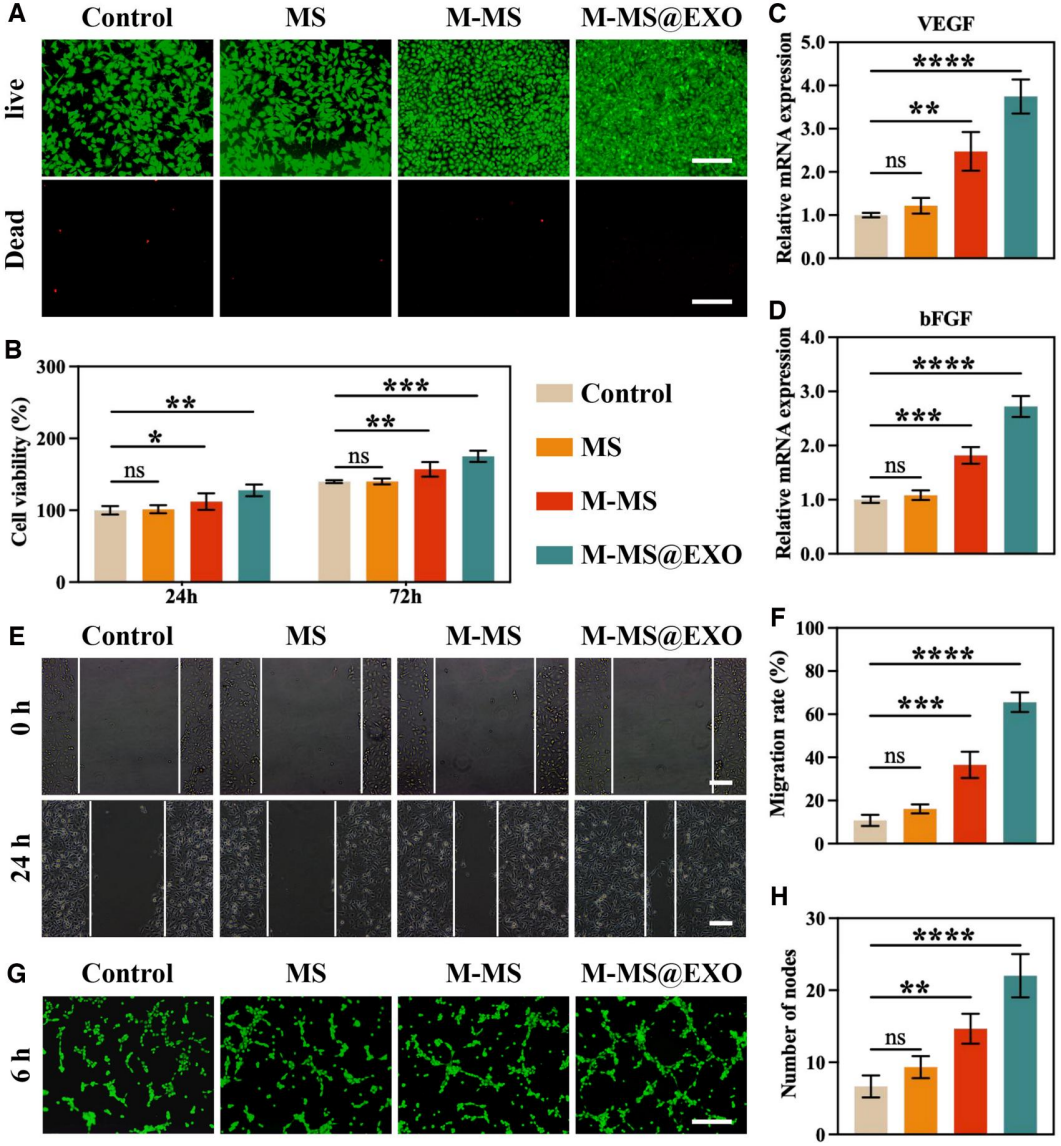
(**A**) Live/dead staining of HUVECs treated with MS, M-MS, M-MS@EXO NPs for 24 h. Scale bars: 100 µm. (**B**) Cell vability of HUVECs cultured with MS, M-MS, M-MS@EXO NPs for 24 and 72 h. (**C** and **D**) Relative mRNA expression of angiogenic genes VEGF and bFGF. (**E**) Scratch wound healing tests on the migration of HUVECs cultured with different NPs from 0 to 24 h. Scale bars: 50 µm. (**F**) Quantification of cell migration rate. (**G**) Tube formation of HUVECs cultured with different NPs for 4 h. Scale bars: 100 µm. (**H**) Quantification of number of nodes. *P < 0.05, **P < 0.01, ***P < 0.001, ****P < 0.0001.

In the investigation of the effects of M-MS@EXO *in vitro*, CCK-8 assays and live/dead staining were first conducted to assess the potential cytotoxicity of M-MS@EXO. The results illustrated that M-MS@EXO elicited no discernible cytotoxic effects on HUVECs, demonstrating excellent biocompatibility, which is crucial for therapeutic applications involving cellular environments (Figure 3A). Rather than causing harm, M-MS@EXO manifested a pronounced enhancement in the proliferative capacity of HUVECs, suggesting a beneficial role in supporting vascular health (Figure 3B). Additionally, quantitative PCR analysis was performed to measure the mRNA expression levels of key angiogenic markers, VEGF and bFGF. Both them are critical regulators of angiogenesis, playing essential roles in promoting endothelial cell proliferation, migration and new blood vessel formation. The results demonstrated a significant upregulation of both markers in HUVECs treated with M-MS@EXO NPs, further reinforcing the compound’s pro-angiogenic potential (Figure 3C and D).

Building on these encouraging findings, subsequent migration assays and *in vitro* Matrigel tube formation assays demonstrated that M-MS@EXO significantly enhanced both the migratory capacity and angiogenic potential of HUVECs. This was evident through an accelerated wound closure rate observed in scratch assays, where cells treated with M-MS@EXO migrated more swiftly to fill the gaps, showcasing the potential of promoting vascular repair and regeneration (Figure 3E and F). Furthermore, the increased tubular network complexity noted in Matrigel-based angiogenesis assays further confirmed the pro-angiogenic effects of M-MS@EXO, indicating its role in facilitating the formation of new blood vessels (Figure 3G and H). Therefore, these results collectively underscore the significant therapeutic potential of M-MS@EXO in enhancing vascular integrity and functionality. By promoting endothelial cell activity and supporting angiogenesis, M-MS@EXO plays a crucial role in improving vascular function following flap surgery, particularly in conditions marked by insufficient blood supply and chronic inflammation.

### M-MS@EXO NPs inhibited the release of inflammatory factors from macrophages in vitro

**Figure 4.**
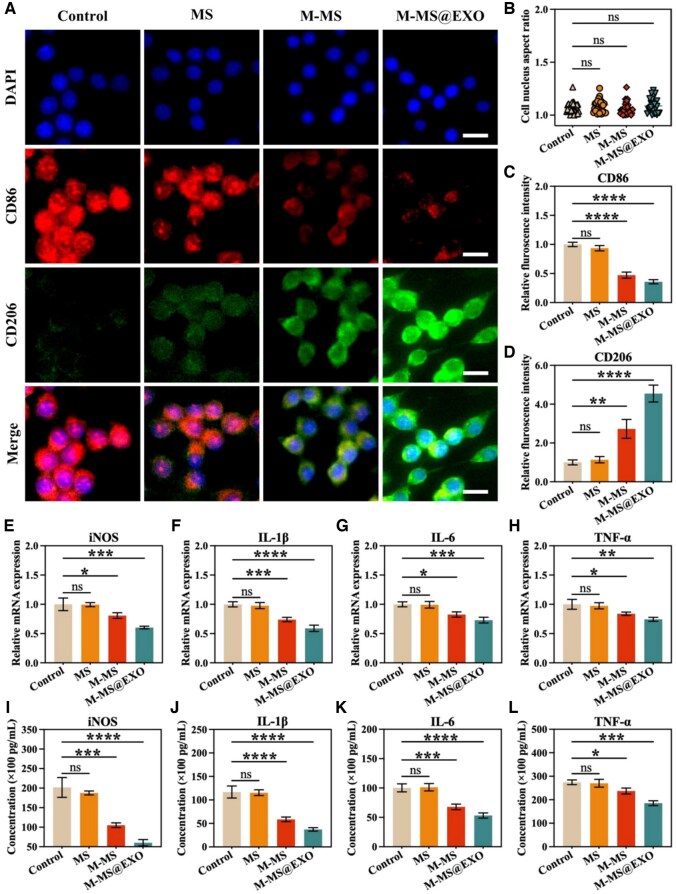
(**A**) Immunofluorescence (IF) staining of nucleus, CD86 and CD206 of RAW264.7 cells on Day 2. Scale bars: 10 µm. (**B**) Quantitative cell nucleus aspect ratio of macrophages based on IF images. (**C** and **D**) Quantitative fluorescence intensity of CD86 and CD206 of macrophages based on IF images. (**E**–**H**) Relative mRNA expression of iNOS, IL-1β, IL-6, TNF-α. (**I**–**L**) ELISA analyses of iNOS, IL-1β, IL-6, TNF-α. *P < 0.05, **P < 0.01, ***P < 0.001, ****P < 0.0001.

Excessive inflammatory responses can significantly disrupt the processes of angiogenesis, and thus, contribute to a reduced survival rate of multi-territory perforator flaps, highlighting the critical need to address inflammation in these surgical contexts. Our research findings demonstrate that the engineered stem cell exosomes, M-MS@EXO, exert a profound regulatory effect on macrophage polarization, as evidenced by a significant downregulation of CD86 expression alongside an upregulation of CD206 expression in macrophages (Figure 4A–D). This shift in surface marker expression indicates that M-MS@EXO effectively inhibits the polarization of macrophages toward the pro-inflammatory M1 phenotype, which is typically associated with increased inflammatory responses and tissue damage. Simultaneously, it promotes the transition to the anti-inflammatory M2 phenotype, fostering a more favorable microenvironment for healing. The ability of M-MS@EXO to modulate these cellular processes plays a vital role in curbing excessive inflammatory progression, contributing to improved vascular regeneration outcomes in the flap model. Further systematic experimental validation has revealed that M-MS@EXO markedly suppresses the expression of various M1 macrophage-associated pro-inflammatory mediators at the mRNA levels. Specifically, Figure 4E–H show a significant downregulation of inducible iNOS, IL-1β, IL-6 and TNF-α, demonstrating transcriptional inhibition of these inflammatory mediators. In addition, the ELISA results shown corroborate these findings, revealing a significant decrease in the secreted levels of iNOS, IL-1β, IL-6 and TNF-α from macrophages treated with M-MS@EXO (Figure 4I–L).

### Transcriptomic analysis shows M-MS@EXO-induced pathways of macrophages

**Figure 5.**
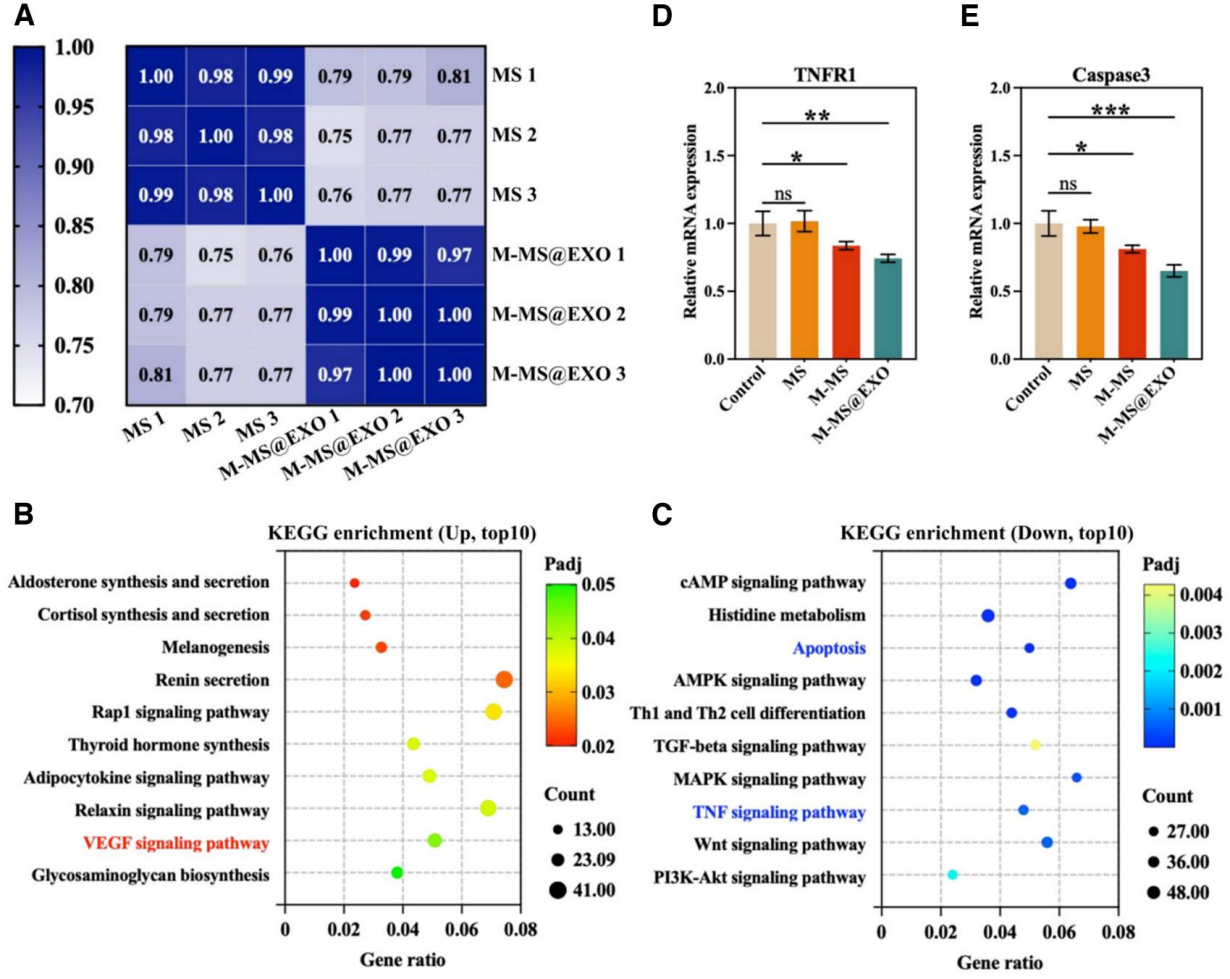
Bioinformatic analysis of macrophage gene expression induced by M-MS@EXO NPs. (**A**) Heatmap of Pearson correlation between samples. (**B** and **C**) Upregurated and down-regulated KEGG enrichment pathways of MS versus M-MS@EXO. (**D** and **E**) Relative mRNA expression of TNFR1 and Caspase3. *P < 0.05, **P < 0.01, ***P < 0.001.

To understand how M-MS@EXO NPs activate macrophage polarization, we performed transcriptomic analysis on macrophages cultured with M-MS@EXO and MS. We first assessed the stability of the samples by conducting a Pearson correlation analysis, indicating a significant degree of differential gene expression (Figure 5A). To analyze potential signaling pathways, we conducted a Kyoto Encyclopedia of Genes and Genomes (KEGG) analysis. The top-enriched upregulated KEGG pathway is shown in Figure 5B, revealing an upregulation of the VEGF signaling pathway, which is known to be associated with vascular regeneration. This finding strongly correlates with the previous observation that M-MS@EXO NPs promote angiogenesis. VEGF comprises a complex mixture of structural and functional macromolecules that play a crucial role in vascular morphogenesis, as well as in maintaining cell and tissue structure and function.

In contrast, pathways associated with the activation of M1 macrophages, particularly the TNF signaling pathway, were downregulated following M-MS@EXO NPs treatment (Figure 5C). Additionally, the apoptosis pathway was also downregulated. Examination of the mRNA levels of TNFR1, a key component of the TNF pathway and Caspase3, a crucial executor in the apoptosis pathway, confirmed these findings (Figure 5D and E). The downregulation of both pathways indicates that M-MS@EXO NPs possess significant anti-inflammatory advantages, supporting future *in vivo* studies on the vascular microenvironment in diabetic conditions.

### M-MS@EXO NPs alleviated perforator flap necrosis in type 2 diabetic rat

**Figure 6.**
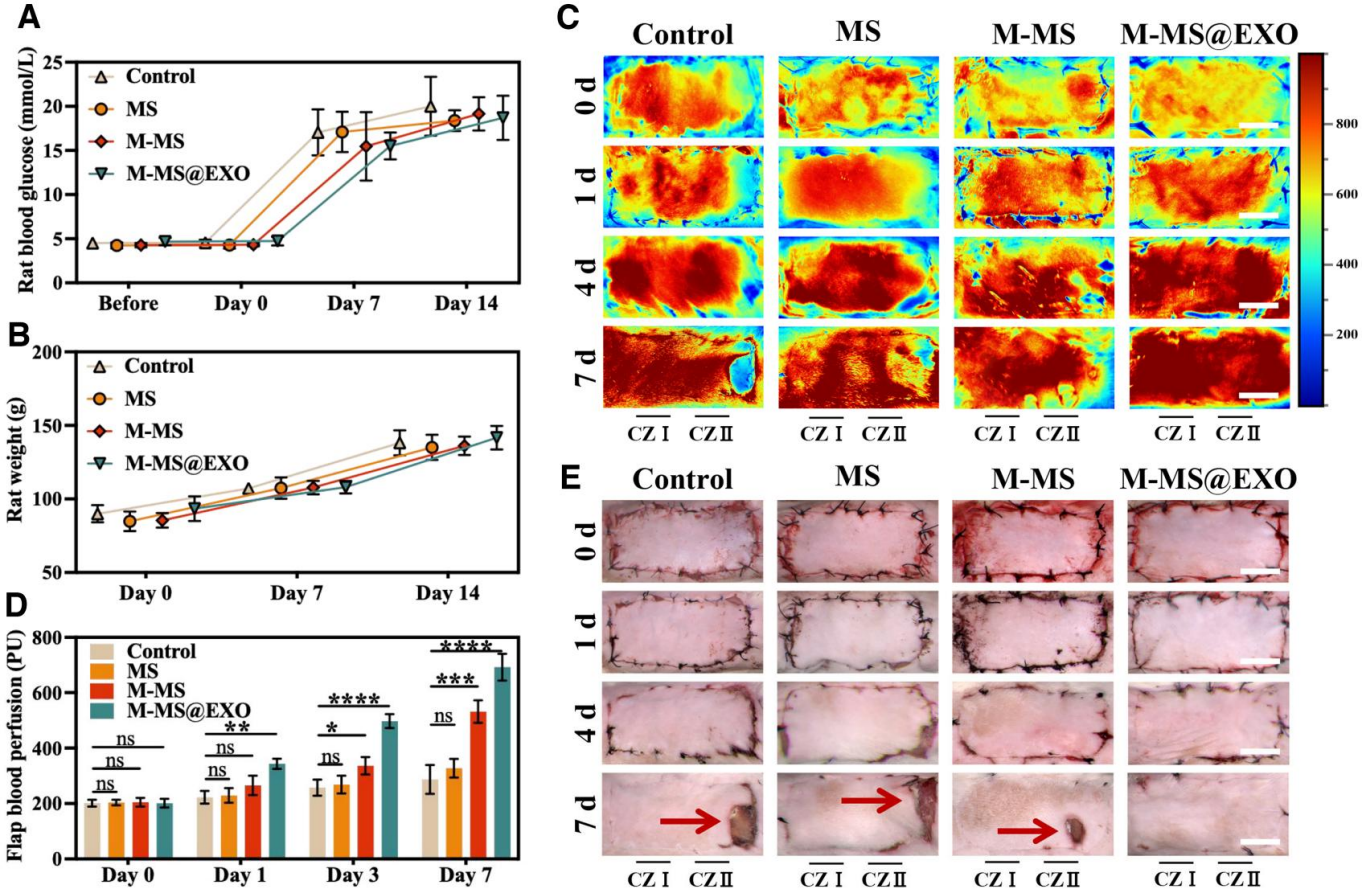
(**A**) Rat lood glucose and (**B**) weight measured at several time points: before, after, on Day 7 and on Day 14 of STZ injection. (**C**) Laser speckle images at Day 0, Day 1, Day 3, Day 7 postsurgery. Choke zone I was abbreviated as CZI, while Choke zone II was abbreviated as CZII. Scale bars: 2 cm. (**D**) Blood perfusion in the Choke zone II of flap based on laser speckle images. (**E**) Flap images at Day 0, Day 1, Day 3, Day 7 postsurgery. Scale bars: 2 cm. *P < 0.05, **P < 0.01, ***P < 0.001, ****P < 0.0001.

The preceding work constituted the foundation for the execution of *in vivo* experimentation. The successful modeling of type 2 diabetic rats was confirmed by an increase in blood glucose and changes in body weight (Figure 6A and B). To investigate the potential preventive effects of M-MS@EXO NPs on perforator flap necrosis associated with diabetes mellitus, we established a type 2 diabetic rat model of multi-territory perforator flap and systematically evaluated blood perfusion dynamics at postoperative Days 0, 1, 4 and 7. Specifically, we aimed to determine whether M-MS@EXO NPs exert protective effects against perforator flap necrosis induced by diabetes. In this context, we closely monitored blood perfusion levels in the rat model at various postoperative intervals (Figure 6C). The experimental findings demonstrated that M-MS@EXO intervention significantly ameliorated flap necrosis compared to untreated controls, highlighting its efficacy in enhancing vascular health in diabetic conditions. Notably, the intervention resulted in remarkable improvements in hemodynamic parameters within Choke zone II, where quantitative analysis revealed statistically superior maintenance of blood flow relative to the control group (Figure 6D). In the control group, no necrosis was observed macroscopically on Day 4 (Figure 6E). However, the blood flow perfusion showed low perfusion in the distal region, which indicated a deteriorating condition that resulted in flap necrosis and tissue loss on Day 7. In contrast, M-MS@EXO NPs exhibited high perfusion in the distal region on Day 4, suggesting that flap healing was proceeding normally. As expected, the M-MS@EXO group demonstrated complete healing of the flaps on Day 7. The distal necrosis after flap surgery led to a loss of flap integrity, resulting in the absence of skin and soft tissue at the necrotic site. This open wound, due to prolonged exposure, significantly increased the risk of bacterial infection. These results underscore the promising therapeutic potential of M-MS@EXO NPs in mitigating distal necrosis of the flap, ultimately contributing to improved outcomes in surgical applications involving vascularized tissue in diabetic patients.

### M-MS@EXO NPs reduced the production of inflammatory factors and suppresses oxidative stress response in vivo

**Figure 7.**
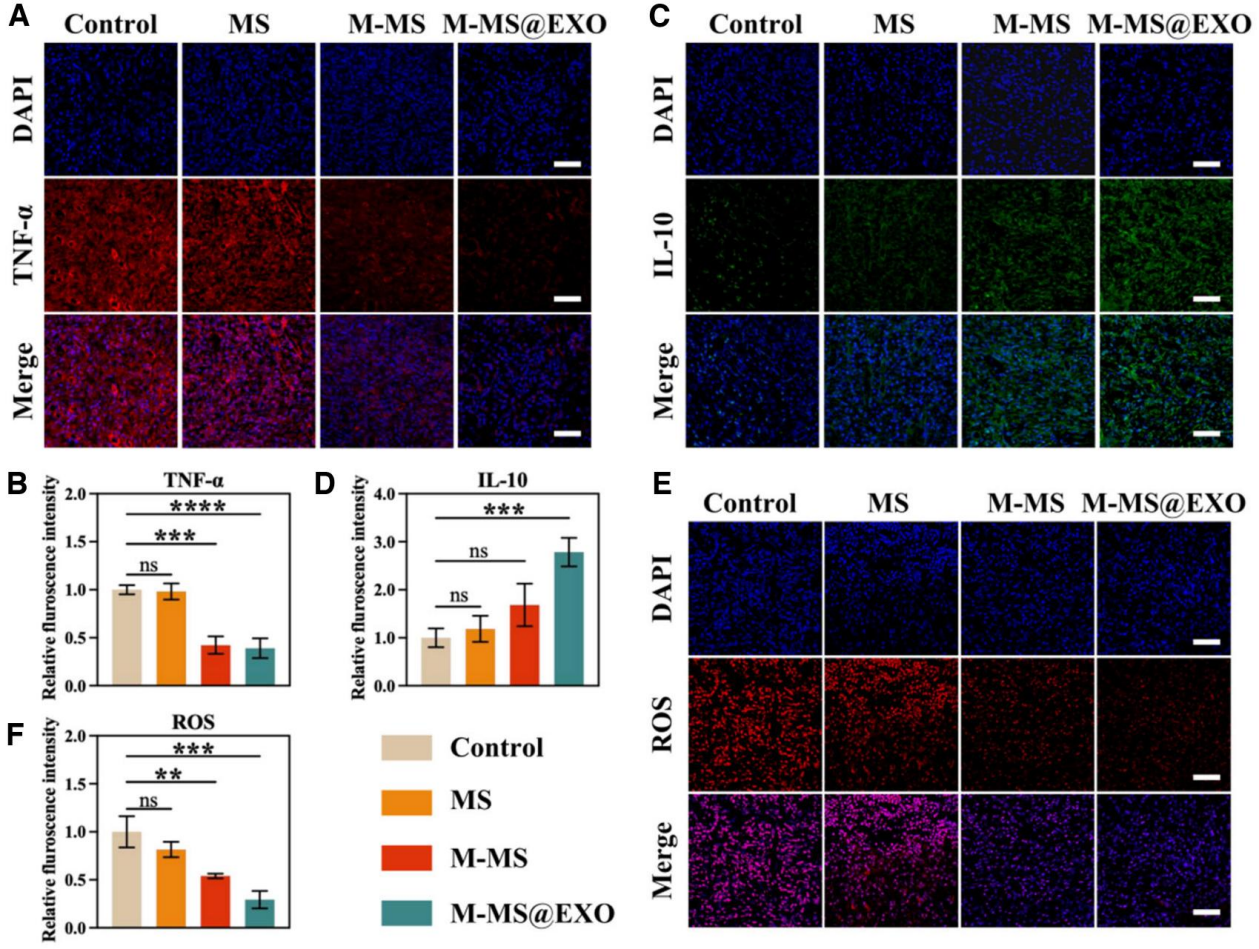
(**A**) IF staining of nucleus, TNF-α and merge of the Choke zone II at Day 3 postsurgery. Scale bars: 200 µm. (**B**) Quantitative fluorescence area of TNF-α based on IF images. (**C**) IF staining of nucleus, IL-10 and merge of the Choke zone II at Day 3 postsurgery. Scale bars: 200 µm. (**D**) Quantitative fluorescence area of IL-10 based on IF images. (**E**) IF staining of nucleus, ROS and merge of the Choke zone II at Day 3 postsurgery. Scale bars: 200 µm. (**F**) Quantitative fluorescence area of ROS based on IF images. **P < 0.01, ***P < 0.001, ****P < 0.0001.

The survival of distal tissues in perforator flaps is critically dependent on the pathophysiological status of Choke zone II. As demonstrated in Figure 7A and B, M-MS@EXO administration significantly suppressed the release of the pro-inflammatory cytokine TNF-α. However, it concurrently enhanced the production of the anti-inflammatory cytokine IL-10 within this critical region (Figure 7C and D). Furthermore, a marked attenuation of oxidative stress responses was observed in M-MS@EXO-treated specimens, as evidenced by reduced lipid peroxidation and restored antioxidant enzyme activities (Figure 7E and F). This dual modulation of inflammatory homeostasis and oxidative balance substantially decreased the probability of excessive inflammatory cascades, thereby improving microcirculatory perfusion through vasodilation and reduced endothelial activation. The observed enhancement of hemodynamic parameters in Choke zone II establishes a favorable microenvironment for cellular metabolism, ultimately providing a robust biological foundation for promoting tissue viability in the distal portion of perforator flaps.

### M-MS@EXO NPs promoted vascular regeneration in vivo

**Figure 8.**
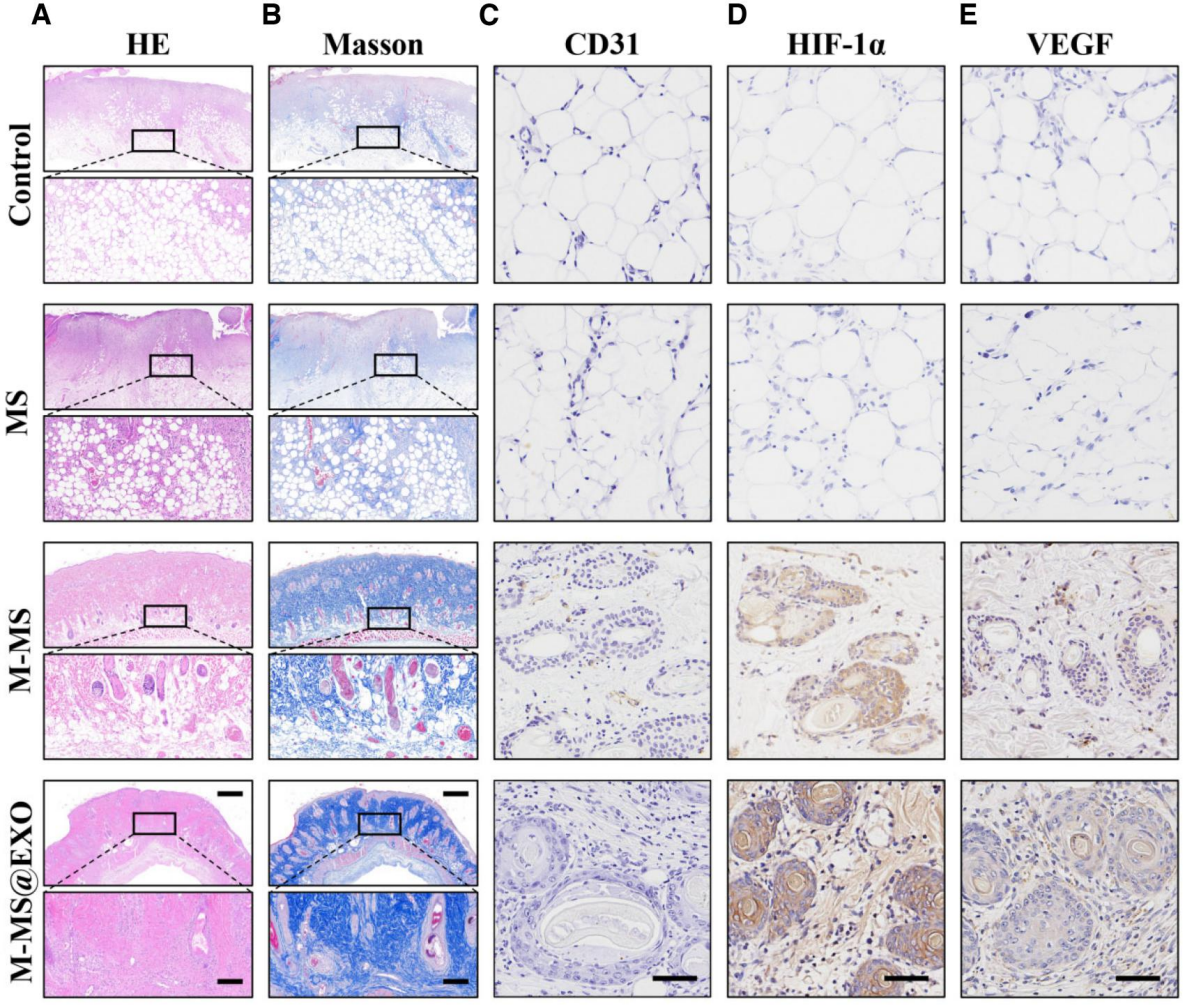
(**A**) H&E staining and (**B**) Masson staining for the Choke zone II of flap at Day 7 postsurgery. Scale bars: 1 mm in low-magnification and 200 µm in high-magnification. Immunohistochemical staining of (**C**) CD31, (**D**) HIF-1α and (**E**) VEGF of the Choke zone II zone at Day 7 postsurgery. Scale bars: 200 µm.

Histological assessment of Choke zone II on Day 7 after surgery revealed enhanced vascular regeneration in the M-MS@EXO group, as indicated by the presence of denser fibrous and collagenous tissue (Figure 8A and B). In contrast, the control group exhibited excessive foam-like necrotic tissue and sparse fibre and collagen. Figure 8C–E showed histochemical staining for key markers, including CD31, HIF-1α and VEGF within the Choke zone II. These stains highlighted essential markers involved in angiogenesis and vascular function, allowing for the assessment of vascularization associated with the tissue’s response following surgical intervention. The IHC images revealed a greater number of HIF-1α, VEGF and eNOS-positive cells in the M-MS@EXO group, followed by the M-MS group. Therefore, the findings suggest that M-MS@EXO NPs play a significant role in promoting angiogenesis of Choke zone IIin the multi-territory perforator flap.

Additionally, to explore the metabolism of M-MS@EXO NPs in rat and determine whether it can cause damage to the metabolic organs in the body, we conducted pathological studies on the heart, liver, spleen, kidney and lung of experimental rat. As shown in Supplementary Figure S2, H&E staining showed no pathological abnormalities between each group, thus, indicated the therapeutic M-MS@EXO NPs had no toxic effect on the metabolic organs of experimental rats.

## Discussion

The multi-territory perforator flap is a powerful tool for repairing complex diabetic wounds [34]. However, the high-glucose condition engenders unremitting oxidative stress within the flap microenvironment, culminating in endothelial cell deterioration and flap necrosis [35, 36]. The resolution of necrosis is contingent upon the restoration of blood supply and functionality to the Choke zone. Choke vessels act as a bridge connecting adjacent perforator vessels, serving as the sole pathway for blood supply in cross-regional perforator flaps. They play a crucial role in ensuring the survival of these flaps. Studies have shown that flap necrosis frequently occurs in the hemodynamic region and potential donor region, specifically in the areas known as the Choke zone I and Choke zone II [37]. Necrosis in the distal region of the multi-territory perforator flap is related to Choke zone II, as Choke zone II represents the watershed area with increased vascular resistance [38]. In recent years, exosome-based therapies for vascular regeneration have gained increasing research attention. However, to overcome the inherent limitations of natural exosomes previously discussed, we proposed an engineered exosome platform incorporating metformin-loaded mesoporous silica nanoparticles. This innovative design synergistically combined the biological compatibility of exosomes with the drug delivery advantages of mesoporous silica nanoparticles, while addressing the pharmacokinetic challenges associated with conventional exosome therapy.

Recently, stem cell-derived engineered exosomes have gained significant attention as promising tools for various medical applications due to their ability to incorporate numerous bioactive molecules [39, 40]. These exosomes leverage the intrinsic properties of natural exosomes, which facilitate intercellular communication by transporting essential components such as proteins, mRNAs and miRNAs [41–43]. The key advantages of engineered exosomes include excellent tissue compatibility, which minimizes immune responses and promotes better integration with host tissues. Additionally, their multifunctional capability allows for the simultaneous delivery of multiple therapeutic agents, making them safer and more cost-effective compared to conventional delivery methods. We were inspired to introduce engineered exosomes for the treatment of complex diseases such as diabetic perforator flaps, which often face significant challenges of impaired angiogenesis and chronic inflammation. The M-MS@EXO NPs exhibited favourable biocompatibility and pro-cellular proliferation in the study, thereby substantiating the capacity of engineered exosome to protect and restore cellular function under diabetic conditions.

The viability of a flap is contingent upon the perfusion status of the local tissue [44], which can be quantitatively assessed through laser Doppler flowmetry. Our research demonstrated that, compared to the control group, the mean blood perfusion in the Choke zone II of the perforator flap was elevated on Day 4 post M-MS@EXO NPs intervention, with a more pronounced enhancement observed on Day 7. These findings suggest that M-MS@EXO effectively improves the microcirculatory efficiency of the flap vasculature, as well as the microvascular density of arterial perforators in the Choke zone II. Besides, we discovered that vascular endothelial cells treated with the M-MS@EXO NPs showed enhanced angiogenesis compared to the control group *in vitro*. Histological assessment of the flap Choke zone II on Day 7 after surgery also revealed that the M-MS@EXO NPs significantly promoted VEGF expression in endothelial cells, which played a crucial role in angiogenesis [45].

Excessive inflammation can impair the healing of perforator flaps. Chronic inflammation may lead to increased vascular permeability, which can result in tissue edema and compromised perfusion, reducing oxygen and nutrient delivery to the flap [46]. It can also increase the risk of flap congestion, necrosis and infection. If pro-inflammatory cytokines such as TNF-α and IL-6 are overproduced, they can lead to tissue damage and fibrosis, ultimately impairing the healing process [47–49], which in our research was demonstrated be suppressed through immunofluorescence assay compare to the control. Via immunofluorescence analysis, we found that macrophage cell lines subjected to M-MS@EXO NPs intervention exhibited significantly reduced CD86 expression, which can interact with CD28 [50] and CTLA-4 receptors on T cells, providing necessary co-stimulatory signals that support T cell activation, proliferation and IL-2 production [51], ultimately contributing to the propagation of the inflammatory response, compared to the control group. In contrast, M-MS@EXO NPs treatment in the experimental group significantly promoted the expression of CD206 which plays a critical role in the immune system by facilitating the clearance of unwanted mannose-containing glycoproteins [52], thereby aiding immune cells in maintaining immune homeostasis and restraining inflammatory progression. Interestingly, transcriptome sequencing showed that M-MS@EXO NPs downregulated the TNF and apoptosis signaling pathways, and the downregulation of key genes supported its anti-inflammatory effects, demonstrating the anti-inflammatory and anti-apoptotic mechanisms of this engineered exosomes. The pathological staining analysis of Choke zone II indicated that the condition of the local flap tissue on the 7th day postintervention was markedly superior to that of the control group. Furthermore, a marked attenuation of oxidative stress responses was observed in M-MS@EXO NPs group, as evidenced by reduced peroxidation. These findings highlight the significant efficacy of the M-MS@EXO NPs in promoting improved tissue health and mitigating pathological changes.

*In vitro* and *in vivo* results clearly demonstrated that the combination of mesoporous silica NPs and exosomes enhanced blood perfusion in the Choke zone II region and ultimately improved the survival rate of multi-territory perforator flaps by modulating inflammatory responses and promoting neovascularization. However, the underlying mechanisms require further elucidation, and it is worth noting that physiological discrepancies between rodent models and humans may lead to divergent responses to the M-MS@EXO NPs. In future designs of exosomes, engineered exosomes loaded with neurotrophic factors (e.g. BDNF), could be targeted for diabetic neuropathy (e.g. sensory loss) to simultaneously promote nerve axon regeneration and vascular network reconstruction.

## Conclusion

This study explores the role of M-MS@EXO NPs in promoting the survival of multi-territory perforator flaps in diabetes, focusing on regulating anti-inflammatory responses and enhancing angiogenesis. The engineered exosomes were developed by encapsulating mesoporous silica nanoparticles (MSNs) to facilitate the release of metformin, which effectively modulates inflammatory cytokine levels and improves the vascular niche in diabetic tissues. In addition, M-MS@EXO NPs significantly reduced the expression of pro-inflammatory mediators and promoted the transition of macrophages from the M1 to the M2 phenotype by inhibiting TNF signaling pathway, thereby alleviating excessive inflammation and enhancing vascular regeneration. In the type 2 diabetic rat model, M-MS@EXO NPs not only reduced flap necrosis but also improved blood perfusion dynamics in the Choke zone. These findings indicate that engineered exosomes can effectively mitigate the inflammatory response and promote neovascularization, providing a promising therapeutic strategy for improving multi-territory perforator flap survival in the diabetic microenvironment. M-MS@EXO NPs demonstrate the ability of dual regulation of inflammation and angiogenesis to improve tissue viability and provide new directions for exosome engineering in complex wound healing.

## Funding

This work was supported by the supported by the National Natural Science Foundation of China (No. 82072440), the Science and Technology Innovation Cultivation Fund of Zhongnan Hospital of Wuhan University (No. CXPY2023028), Excellent Doctor Fund Project of Zhongnan Hospital of Wuhan University (No. ZNYB2022015), Natural Science Foundation of Hubei Province (No. 2024AFD167) and the China Postdoctoral Science Foundation (No. 2023M742701).

## Supplementary Material

rbaf075_Supplementary_Data
