# Supplementary material for: Engineering stem cell exosomes promotes the survival of multi-territory perforator flap in diabetes via regulating anti-inflammatory and angiogenesis
Source: Regen Biomater. 2025 Jul 24;12:rbaf075. doi: 10.1093/rb/rbaf075 (PMC12364439; doi:10.1093/rb/rbaf075)
Supplement: rbaf075_Supplementary_Data [file rbaf075_supplementary_data.doc]

**Supporting Information**

**Engineering stem cell exosomes promotes the survival of multi-territory perforator flap in diabetes via regulating anti-inflammatory and angiogenesis**

Chao Sun^1,^**^†^**, Junwei Su^1,^**^†^**, Zheng Wang^1^, Changjiang Liu^1^, Xinzeyu Yi^1^, Weimin Chen^1,*^, Dong Zhang^1,*^, and Aixi Yu^1,*^

^1^Department of Orthopedics Trauma and Microsurgery, Zhongnan Hospital of Wuhan University, Wuhan, Hubei 430071, China

*Corresponding authors.

E-mail addresses: cwm68@163.com(W. C.); zhangdongemail@whu.edu.cn (D. Z.); yuaixi@whu.edu.cn (A. Y.)

**^†^**These authors are co-first authors.

**Table S1.** The forward and reverse primer sequences for RT-PCR performance.

| Primer | Forward | Reverse |
| --- | --- | --- |
| VEGF | CTCCACCATGCCAAGTGGTC | GCAGTAGCTGCGCTGATAGA |
| bFGF | CGGCTGTACTGCAAAAACGG | GATGTGAGGGTCGCTCTTCTCC |
| iNOS | GCTCTAGTGAAGCAAAGCCCA | TCTCTCCACTGCCCCAGTTT |
| IL-1β | TGGCAACTGTTCCTG | GGAAGCAGCCCTTCATCTTT |
| IL-6 | AGTTGCCTTCTTGGGACTGA | CAGAATTGCCATTGCACAAC |
| TNF-α | CGCTCTTCTGTCTACTGAACTTCGG | GTGGTTTGTGAGTGTGAGGGTCTG |
| TNFR1 | ACTTGGGAGTGACTGTCCGAGC | GGAAGTGTGTCTCACTACGGTA |
| Caspase3 | GAAACTCTTCATCATTCAGGCC | GCGCGTGAGAATGTGCATAAAT |


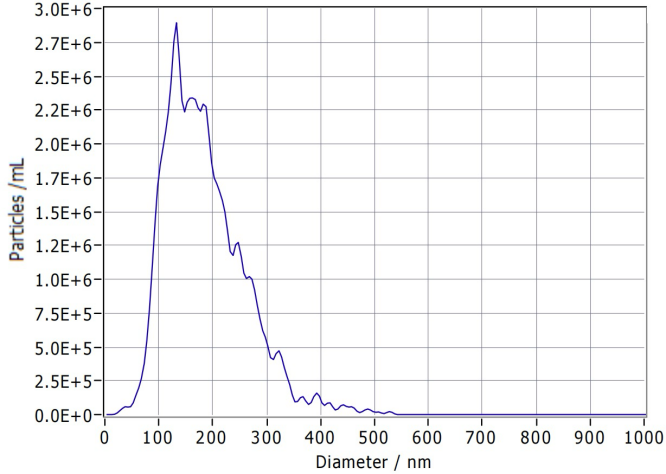


**Figure S1.** (A) Particle size distribution of BMSCs exosomes measured by DLS.


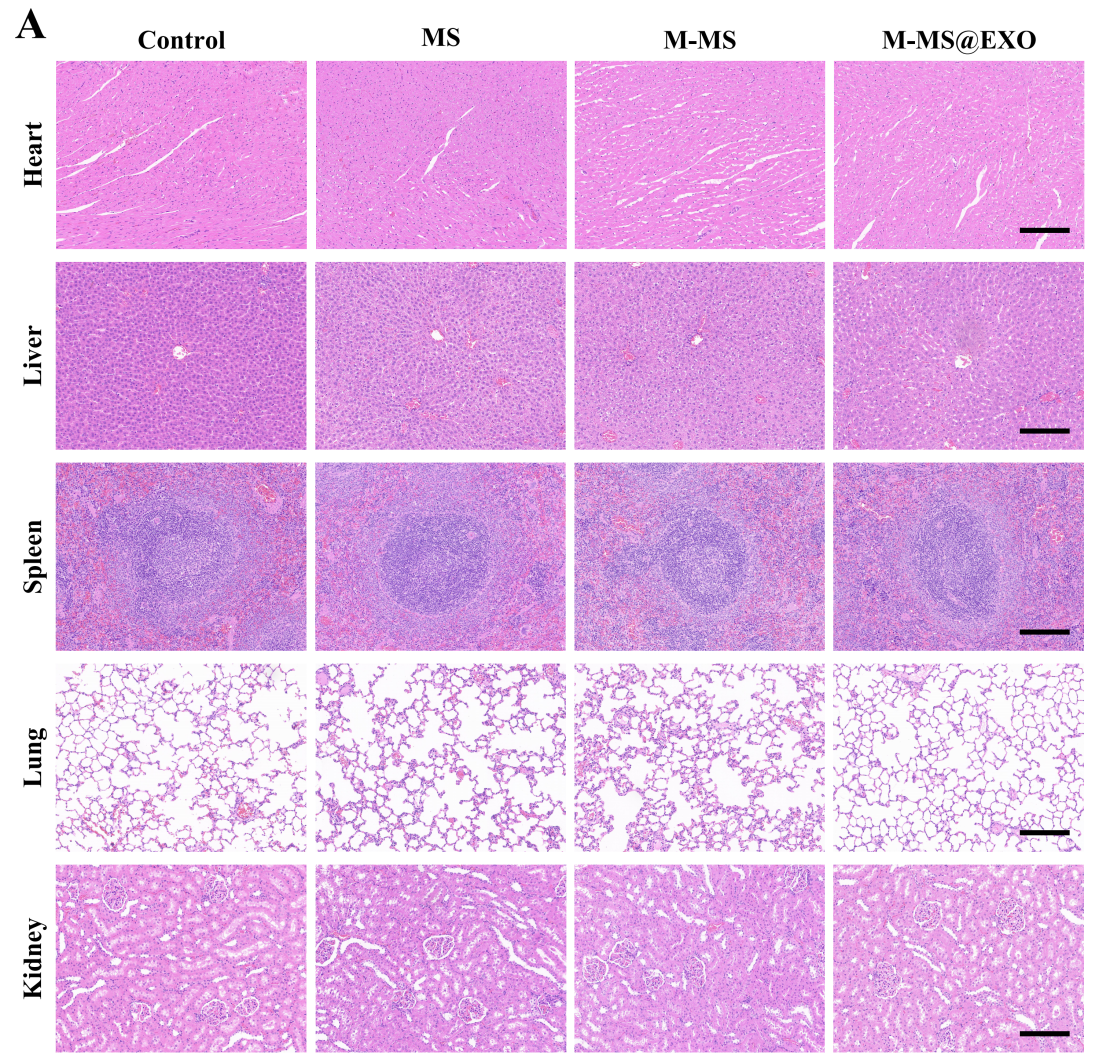


**Figure S2.** (A) H&E staining for heart, liver, spleen, lung, and kidney tissues of the Control, MS, M-MS, and M-MS@EXO groups at day 7 post-surgery. Scale bars: 200 µm.
